# Temperature Measurement of Hot Airflow Using Ultra-Fine Thermo-Sensitive Fluorescent Wires †

**DOI:** 10.3390/s22093175

**Published:** 2022-04-21

**Authors:** Shumpei Funatani, Yusaku Tsukamoto, Koji Toriyama

**Affiliations:** Department of Mechanical Engineering, University of Yamanashi, Yamanashi 400-8510, Japan; g21dts03@yamanashi.ac.jp (Y.T.); toriyama@yamanashi.ac.jp (K.T.)

**Keywords:** LIF, temperature measurement, airflow, fluorescent paint

## Abstract

In this paper, we propose a temperature measurement method that uses ultrafine fluorescent wires to reduce the wire diameter to a much lesser extent than a thermocouple. This is possible because its structure is simple and any material can be used for the wire. Hence, ultrafine wires with a Reynolds number of less than 1.0 can be selected. Ultra-fine wires less than 50 µm in diameter were set in the test volume. The wire surfaces were coated with fluorescent paint. The test volume was illuminated using an ultraviolet light-emitting diode. The paint emits very tiny, orange-colored fluorescent light with an intensity that changes with the temperature of the atmosphere. The experimental results showed that the heating/cooling layers were well visualized and the temperature field was well analyzed.

## 1. Introduction

Two- and three-dimensional temperature-distribution-measurement technologies are necessary for the performance evaluation of air-conditioning equipment and heat exchangers. For example, it is possible to measure the temperature distribution of an entire device, identify the heat-generating point, and compare this heat distribution with the heat-resistant temperature of the component.

Thermistors, thermocouples, and platinum resistance thermometers can be used to measure the temperature at a single point. Thermistors are often incorporated into mass-produced products owing to their low cost, whereas thermocouples and platinum resistance thermometers are often used for inspection and performance evaluation, owing to their advantages in high-precision measurement. The measurement principle of a thermocouple is to measure the electromotive force generated by the temperature difference between the two ends of dissimilar metal wires. The sensor can be easily downsized, owing to the simple structure of the sensor part and can be made into a sensor with a small heat capacity and high temperature response.

There is a strong demand from the industrial world for field measurement technologies for the ambient temperature of gases. For example, in the performance evaluation of a heat exchanger, the temperature distribution can be measured by sweeping thermocouples, but this is limited to the comparison of basic physical quantities, such as the average temperature and temperature fluctuation. Thus, sufficient information necessary for designing the heat exchanger cannot be obtained. In addition, there is a problem with the influence of swept thermocouples on flow conditions. As a countermeasure, it is sometimes possible to increase the safety margin by increasing the size of the heat exchanger. However, such countermeasures cannot be adopted in many cases, owing to restrictions such as the size of the housing and fluid noise. Therefore, there is a great need for non-contact multi-point measurements of the gas temperature distribution.

Liquid crystal thermometry is a field measurement technique for evaluating the temperature in a liquid flow, which combines temperature visualization by suspended particles of liquid crystals and the color image processing technique. The color of the liquid crystals changes quickly when luminated by white light. This change is from colorless to red at low temperatures, green and blue to violet, and colorless at high temperatures. Therefore, the calibration of the optical characteristics of the liquid crystal with variations in temperature is a general approach for quantifying the visualized color images. The liquid crystal works like an optical silicon biosensor for environment monitoring and bio-sensing applications [1]. The variation in the color of a liquid crystal with temperature is complex, which poses a calibration difficulty for determining the temperature from a given set of RGB values. Since the calibration study of liquid crystals by Wilcox et al. [2], various calibration techniques have been investigated in different color spaces [3,4,5]. Dabiri and Gharib [6] proposed the use of the HSI color space to calibrate the color of liquid crystals with varying temperature. Since then, the parameter hue H (degree of color) has been used as a calibration variable for temperature. This calibration technique has been successfully applied to various thermal phenomena [7,8,9]. Subsequently, Fujisawa et al. [8] proposed a hue/intensity calibration technique to improve the accuracy of the hue calibration technique. Furthermore, the application of neural networks to calibrate liquid crystals has been studied in the RGB color space [10]. Such neural-network-based color recognition is used in analysis and evaluation using RGB-D sensors [11]. The merit of using neural networks for liquid crystal calibration is an extension of the measurable temperature range [12]. However, it requires considerably higher computing time than one-parameter calibration techniques, such as hue and hue/intensity. It is common knowledge that the color of the liquid crystal changes not only with temperature but also with the viewing angle relative to the light sheet. This viewing angle effect is important for improving the accuracy of temperature measurements [13,14]. Most temperature measurements in the literature have neglected the viewing angle effect because of the difficulty in analyzing this phenomenon.

Recently, liquid crystal thermometry has been extended to the measurement of three-dimensional temperature fields. Fujisawa and Adrian [15] proposed a scanning light-sheet technique in which a light sheet is traversed and a number of sequential images are captured using a camera at a fixed position, and the three-dimensional structure of thermal plumes over a heated surface is visualized using a reconstruction technique. A similar technique, but with a fixed light sheet and moving target images, has been investigated in a rotating axisymmetric thermal flow [16].

The LIF-measurement method is being developed as a three-dimensional measurement method for temperature distribution in fluids (liquids and gases). If a small amount of a tracer substance is mixed in a fluid and changes color depending on temperature change, the temperature distribution can be measured by capturing the color change as an image and converting the color information into a temperature value [17]. Temporal and spatial variations in the intensity of the excitation light during measurement are sources of measurement error. However, when laser light is used as the excitation light, it is difficult to maintain the intensity of the excitation light constant over time and space. Therefore, the two-color LIF method has been proposed to quantitatively measure scalar quantities based on the ratio of emissions at two different wavelengths [18,19]. In this two-color LIF method, the use of fluorescent molecules with high and low temperature dependence can eliminate the effects of inhomogeneity and temporal variation in the intensity distribution of the excitation light. However, most of the fluorophores used in the LIF method have light-quenching characteristics, and the difference in light quenching characteristics between the two types of fluorophores is an error-causing factor.

We have also studied airflow temperature distribution measurements using a mist of atomized fluorescent dye [20]. In the water temperature distribution measurement, the fluorescent dye is diluted using the water to be measured. Because water mist evaporates easily, the fluorescent dye was dissolved in ethanol, diluted with propylene glycol, which has low volatility, and sprayed. However, this technique has a large error due to non-uniformity in fluorescence intensity because droplets containing the fluorescent dye are sprayed into the gas.

Therefore, in the field of product development, a simple airflow temperature measurement method using thermography was adopted. Because thermography can measure the surface of a solid, a thin strip of paper can be placed in air, and the temperature distribution on the surface of the strip can be measured using thermography. Furthermore, because the temperature of a piece of paper struck by airflow is almost the same as the airflow temperature, we can obtain an approximate measurement of the airflow temperature distribution. However, owing to the low resolution of thermography, it is not possible to capture the temperature distribution on the surface of ultrafine wires that have diameters sufficiently small to render the influence of airflow disturbance negligible.

In this paper, we propose a temperature measurement method that uses ultrafine fluorescent wires to reduce the wire diameter to a much lesser extent than that of a thermocouple. A notable feature of this novel measurement method is that it combines the ease of handling of thermocouple temperature sensors with the non-disturbance of the fluid used in the LIF method [17,18,19,20]. This is possible because the structure of the proposed method is simple, and in this method, various materials can be used for the wire. Hence, ultrafine wires with a Reynolds number less than 1.0 can be selected. The temperature range of this technique is expected to be 0–100 °C (rhodamine B) or 100–750 °C (BAM-B, BAM-G phosphor) [21]. This implies that turbulent flow is not generated downstream of the wire, and the wake is negligible. Furthermore, the required number of wires decreases because the line profile of the temperature can be measured using only one wire. This paper is organized as follows. Section 2.1 describes the principle of temperature measurement using fluorescent dyes, Section 2.2 specifically introduces the properties of the fluorescent dyes used in this study, Section 3 presents specific examples of measurement devices, and Section 4 discusses their measurement uncertainties in detail. A case study of the measurement is presented in Section 5.

## 2. Measurement Methods

### 2.1. LIF Method Using a Fluorescent Dye

The light energy (*I*) [W/m^2^] emitted per unit time by a fluorescent dye in a unit microvolume is proportional to the number of molecules absorbing photons of the excitation light in a unit volume per unit time. $I_{0}$ is the excitation light flux incident on the microvolume [W/m^2^]; *C* is the concentration of the fluorescent dye [g/m^3^]; ∅ is the quantum yield, that is, the ratio of the absorbed excitation light contributing to the fluorescence emission; and ε is the absorption coefficient, that is, the ratio of the light intensity absorbed when the excitation light passes through a solution of unit concentration over a unit length to the intensity of the incident excitation light [m^2^/g]. The luminous flux ($I_{0}$) [W/m^2^], expressed by Equation (1), is incident on a finite volume of a fluorescent dye solution, and the solution is exposed over distance *x* [m]:

(1) $$I=I_{0}C\emptyset \varepsilon$$

The excitation flux at the point of passage ($I_{0}^{t}$) is expressed as follows:

(2) $$I_{0}^{t}=I_{0}e^{-\varepsilon xC}$$

This is known as the Beer–Lambert law, which states that incident light is absorbed and attenuated as it passes through a solution. 

### 2.2. Measurement of Temperature Using the LIF Method

The excitation light intensity ($I_{0}$) is constant, and if the transit distance (*x*) or the concentration is small, $e^{-\varepsilon xC}=1$ (constant), and *I* is proportional to the concentration. The concentration of the fluorescent dye can be measured based on the fluorescence intensity. The two-dimensional distribution can be measured by measuring the fluorescence intensity using a camera. If the fluorescence intensity at a known concentration is obtained in advance, the absolute value of the concentration can be measured.

The quantum yield is temperature-dependent and generally decreases with increasing temperature. This is because higher temperatures tend to cause energy loss due to molecular collisions, internal conversion, and intersystem crossing. However, if the temperature dependence of the absorption coefficient is small, the coefficient is set to zero and the fluorescence intensity is a function of temperature. Rhodamine B, a fluorescent dye that absorbs green light and then emits orange light, is widely used because it has high temperature dependence (approximately −2.3%/K) and is soluble in water.

### 2.3. Characteristics of Rhodamine B

**Figure 1:**
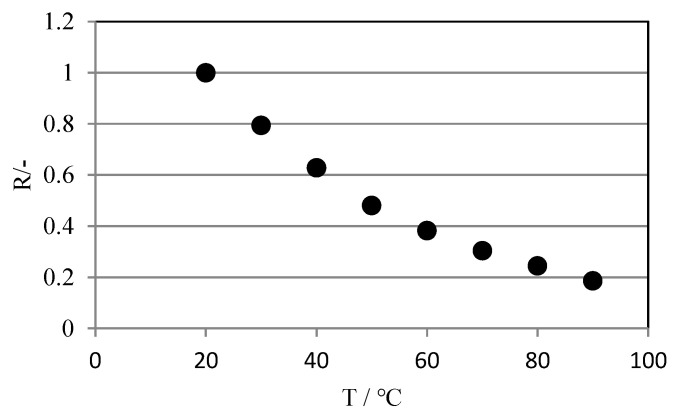
Relationship between the brightness ratio of rhodium B and temperature.

Rhodamine B exhibits a high temperature dependence. Figure 1 shows the relationship between the brightness ratio of rhodamine B and temperature. The fluorescence intensity decreases significantly as the temperature increases. The luminance ratio in the figure is the ratio of the luminance of the RED image (hereinafter referred to as the “R image”), which is the visualized image divided into RGB images, at each temperature to the luminance at 20 °C (set to 1). The temperature measurement range of rhodamine B is approximately 0 to 100 °C.

## 3. Experimental Apparatus and Procedure

**Figure 2:**
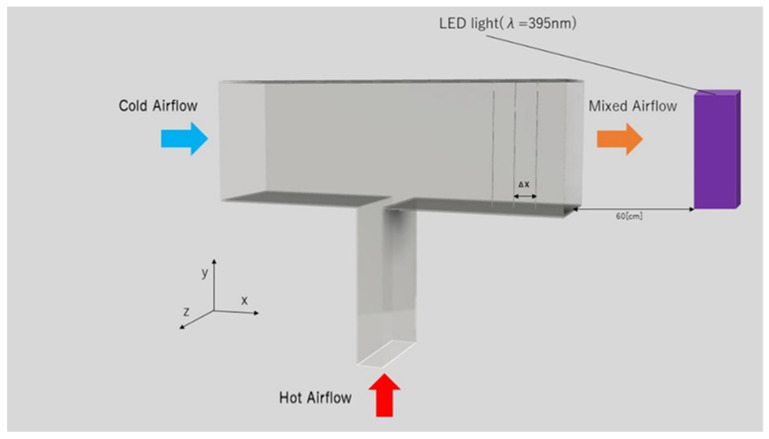
Experimental setup.

Visualized images were obtained when Rhodamine B was painted on the ultrafine line. Visualized images were not obtained when the liquid crystal was painted, owing to the weak reflection of the liquid crystal. Therefore, we decided to perform LIF. Figure 2 shows an evaluation system for the mixing mechanism of cold and hot air of an automobile air-conditioning system model. Cold air was blown from the left side of the device and warm air from the bottom side, and mixed gas was discharged from the right side of the device. For this measurement, several ultrafine stainless wires coated with phosphor were placed in the atmosphere, and the wires were irradiated with light from an ultraviolet (UV) light emitting diode (LED) lamp (center wavelength: 385 nm) to obtain the photographic images necessary for the LIF method. Ultrafine fluorescent wires were placed in the y-direction every ⊿*x* = 10 mm, and visualization images were acquired in the positive z-axis direction using an ultrasensitive digital SLR camera (Nikon D7100). To perform this measurement, the camera must have a high grayscale of at least 10 bits and a sensitivity of at least ISO 6400 (no sensitization mode). An image of the rhodamine B fluorescent wire was taken approximately 500 mm from the wire.

The fluorescent wire for the temperature distribution measurement was made by spraying fluorescent paint mixed with rhodamine B on a stainless-steel wire (50 μm) using a spray coating system. The fluorescent paint consisted of rhodamine B, liquid acrylic, and dichloromethane. The stainless-steel wire was wound at a constant speed. Fluorescent paint was sprayed with an airbrush and dried using a heat gun. By using the coating system, a constant thickness for the fluorescent painting can be maintained.

In this study, the single-color LIF technique [15] using rhodamine B was adopted because the fluorescent wires were fixed in the flow field, and their luminescence was stable. In addition, the influence of quenching by illumination becomes larger using rhodamine 110 for the two-color LIF technique [16,17]. The influence of quenching by illumination becomes almost negligible when using only rhodium B.

**Figure 3:**
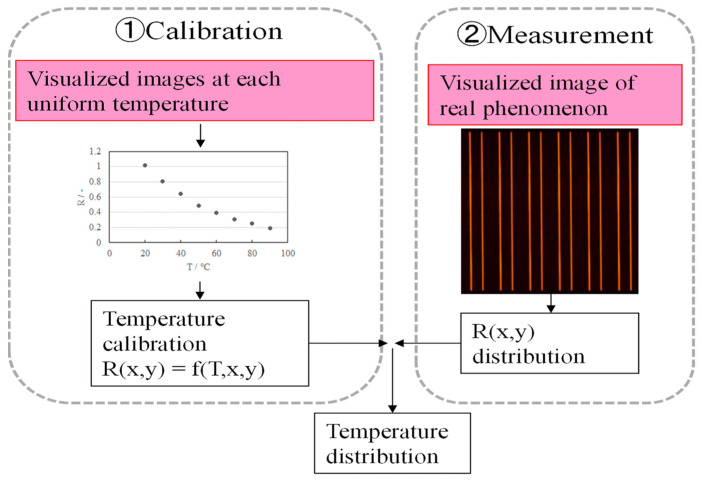
Procedure of temperature evaluation using ultra-fine thermo-sensitive fluorescent wires.

**Figure 4:**
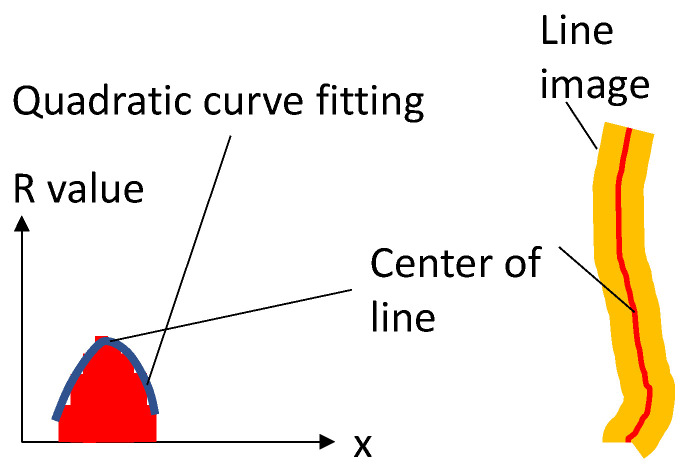
Quadratic curve fitting of the image of the ultrafine wire.

Figure 3 shows a flowchart of the temperature measurement technique. First, the relationship between the temperature and fluorescent illumination was determined, and a calibration curve was generated. Next, the visualized images of the illuminated wires were captured, and the temperature distribution was evaluated using the red images. The incident and scattered UV light became negligible because the color filters used to produce the red images did not transmit UV light. The incident and diffuse reflected UV LED (center wavelength: 385 nm) were completely cut off by the high-pass filter (cutoff wavelength < 450 nm) so that only the fluorescence image could be visualized. The apparent diameter of the ultrafine wire was smaller than one pixel in the visualization image. Therefore, the camera was slightly defocused to increase the apparent diameter. As a result of defocusing the image of the ultrafine wire, the *R* value over the ultrafine wire can be approximated using a quadratic function (Figure 4). The maximum value of this quadratic function was considered to be the *R* value of the wire. Although the thin wire swings slightly under the influence of airflow, the maximum value of the quadratic function remains the same. This measurement system is based on the conventional LIF measurement method [17]. The novelty of this system is the use of ultra-thin wires and the image analysis method tailored to ultra-thin wires (Figure 4).

## 4. Uncertainty Analysis

The feasibility of using the proposed measurement method for flows with non-uniform temperature distribution and dynamic temperature changes must be clarified. In this Section, each source of measurement uncertainty is described in Section 4.1(dynamic temperature change) and Section 4.2 (non-uniform temperature distribution), and a synthesis and summarized discussion on the uncertainty is presented in Section 4.3.

### 4.1. Time Response of Ultrafine Wires

**Figure 5:**
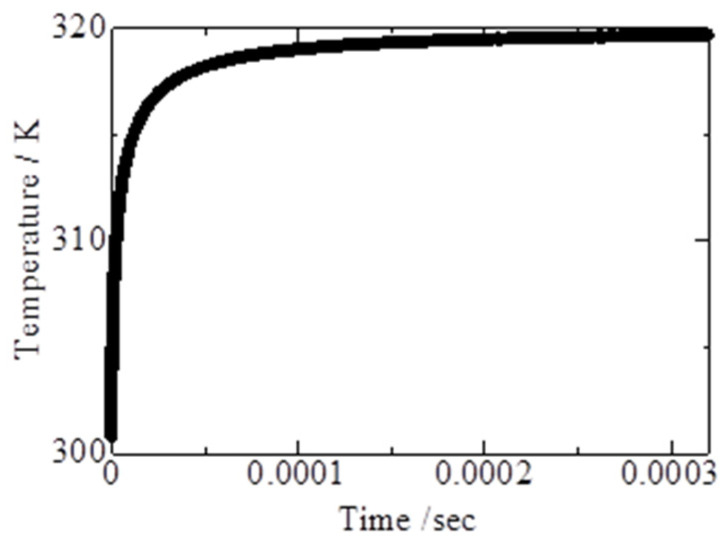
Tracking time of ultrafine wire.

It was necessary to evaluate the tracing accuracy of the ultrafine fluorescent wire because it is related to the temperature of the atmosphere. The temperature variance of a nylon wire surrounded by air was numerically simulated using an unsteady one-dimensional thermal diffusion equation with cylindrical coordinates. Figure 5 shows the change in temperature when the initial temperatures of the wire and atmosphere were 300 K and 320 K, respectively. The tracking time is defined as the time until the temperature difference becomes smaller than 0.1 K. For the considered conditions, the tracking time was less than 1 ms. This evaluation did not consider the influence of convective heat transfer based on the difference between the speeds of the mist and the surrounding air. Therefore, the actual tracing accuracy will be higher than that indicated by this result because the influence of the convective heat transfer results in an improved tracing accuracy. The time variation in the fluorescence intensity was also evaluated. UV light at a power of 30 W was illuminated for 24 h. The results show that the intensity did not change. This shows that the influence of quenching by illumination [15,16] is negligible under this experimental condition.

### 4.2. Spatial Resolution of Temperature Measurement Using Ultrafine Wires

#### 4.2.1. Conditions of the Analysis Area

In this study, the temperature measurement error due to heat conduction in the wire, which is presumed to be the main cause of the uncertainty in the temperature measurement using the new method, was analyzed and verified using a commercially available numerical analysis software, and the measurement error of the new method was evaluated using a numerical analysis. COMSOL Multiphysics, a general-purpose physical simulation software, was used as the numerical analysis software. To evaluate the error between the temperature distribution of the airflow and that of the wire surface, a coupled thermal-fluid analysis was performed.

**Figure 6:**
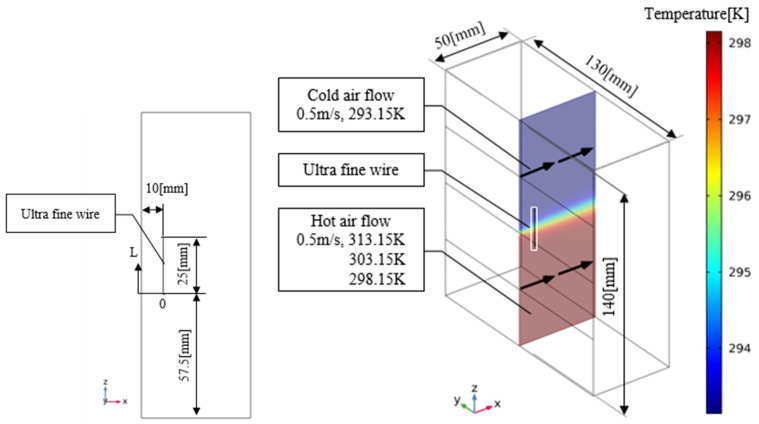
Analysis area and conditions of numerical simulation.

A schematic of the analysis domain is shown in Figure 6. In this study, a rectangular cross-sectional channel 130 mm in width and 140 mm in height was used to simulate the experimental apparatus. To shorten the analysis time, the length of the channel was set to 50 mm, and a 25mm-long cylinder resembling an ultrafine wire crossing the channel in the height direction was placed in the center of the cross-section of the channel, 10 mm from the airflow outlet. The longitudinal coordinate of the surface of the ultrafine wire was represented by the variable *L*, and the lower end of the wire was set to *L* = 0. The fluid in the channel flowed in the direction of the black arrow shown in Figure 6. We assume that a hot air stream flows in the lower half of the channel and a cold air stream flows in the upper half. The temperature gradient of the airflow was generated in the middle of the wire.

**Figure 7:**
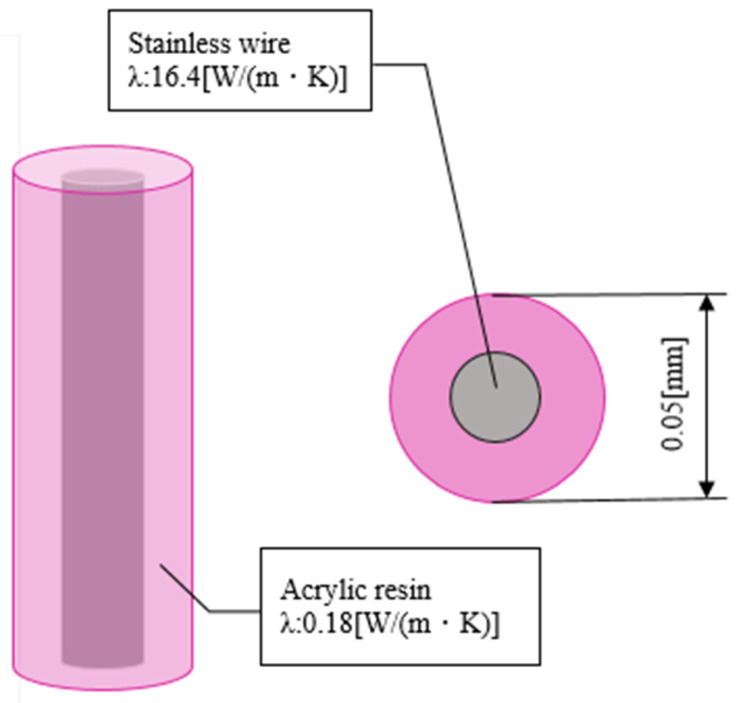
Structure of the ultrafine wire.

The ultrathin wire to be installed in the analysis area was made of a stainless core coated with acrylic resin, similar to the product used in the experiment (Figure 7). The thermal conductivities of stainless steel and acrylic resin were set to 16.4 [W/(m·K)] and 0.18 [W/(m·K)], respectively. In the analysis, the wire diameter was set to 0.05 mm, which is the sum of the diameter of the stainless steel and thickness of the acrylic coating, to evaluate the temperature error under the condition of varying thickness of the acrylic coating (thicknesses: 0, 0.01, and 0.16 mm). The airflow temperatures of the hot and cold sides were set to 313.15 K and 293.15 K, respectively, and the flow velocity was set to 0.5 m/s.

The following table shows the setup conditions for COMSOL. The fluid analysis solver applies turbulence using the Reynolds number of the airflow. The turbulence model uses the *k–ε* model for convergence and computational speed. The equation of the *k–ε* model for an incompressible fluid is as follows:

(3) $$div\left(\rho kU\right)=div\left\{\left(\mu +\frac{\mu_{t}}{\sigma_{k}}\right)gradk\right\}+2\mu_{t}S_{ij}\;\cdot \;S_{ij}-\rho \varepsilon$$

(4) $$div\left(\rho kU\right)=div\left\{\left(\mu +\frac{\mu_{t}}{\sigma_{\varepsilon }}\right)grad\varepsilon \right\}+C_{\varepsilon 1}\frac{\varepsilon }{k}2\mu_{t}S_{ij}\;\cdot \;S_{ij}-C_{\varepsilon 2}\rho \frac{\varepsilon^{2}}{k}$$

(5) $$\mu_{t}=\rho C_{\mu }\frac{k^{2}}{\varepsilon }$$

where *U* is the velocity vector [m/s], *ρ* is the density [kg/m^3^], *k* is the turbulent kinetic energy [m^2^/s^2^], *μ* is the molecular viscosity [Pa s], *μ_t_* is the turbulent viscosity [Pa s], *S_ij_* is the strain rate tensor [/s], and *ε* is the energy dissipation rate [m^2^/s^3^]. The other variables are constants used in the *k–ε* model. We set them to the following values:

(6) $$C_{\mu }=0.09,\;\sigma_{k}=1.00,\;\sigma_{\varepsilon }=1.30,\;C_{\varepsilon 1}=1.44,\;C_{\varepsilon 2}=1.92$$

The heat transfer solver considers the convective heat transfer between the airflow and wire and the heat conduction inside the wire. The energy equation is as follows:

(7) $$\rho C_{p}U\cdot gradT+divq=Q$$

(8) q=−λgradT

where *C_p_* is the constant pressure specific heat [J/(kg·K)], *T* is the temperature [K], *q* is the heat flux [W/m^2^], *Q* is the heat transfer [W/m^3^], and *λ* is the thermal conductivity [W/(m·K)]. There are several examples of similar validations using COMSOL, including the one modeling silicon optical MEMS with miniature monolithic integration in Si-CMOS ICs [22] or the modeling of the fiber-optic sensors [23].

#### 4.2.2. Mesh Partitioning

The mesh used in this analysis was an unstructured tetrahedral mesh, which is a standard mesh used in COMSOL Multiphysics. The mesh size was determined by performing a sensitivity analysis, a common preliminary preparation for CFD analysis. As a result, it was decided that the mesh size in the area away from the ultrafine wire should be 3 mm, and the mesh size inside and around the ultrafine wire should be approximately one-sixth of the wire diameter. The length of one side varies between 5 and 7 μm because the number of wire elements varies depending on the diameter of the stainless-steel wire and thickness of the acrylic coating Therefore, the number of meshes in the entire analysis area varied between 4 and 10 million.

#### 4.2.3. Results of the Numerical Analysis

**Figure 8:**
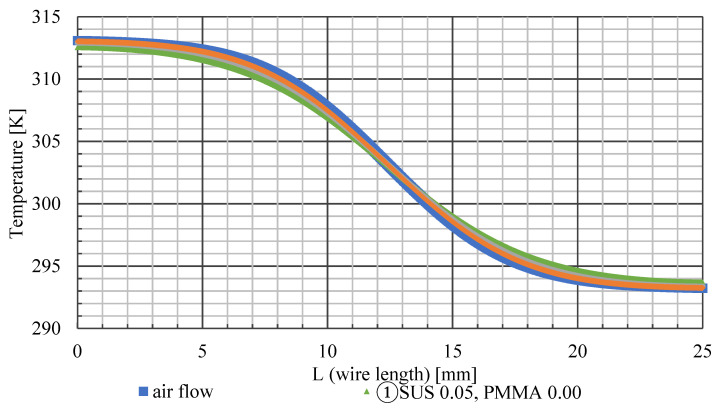
Temperature on wire surface.

Figure 8 shows the temperature distribution of the airflow and surface of the ultrafine wire. The horizontal axis of the graph represents the longitudinal coordinate (*L*) of the ultra-fine wire, and the vertical axis represents the surface temperature of the ultra-fine wire at the longitudinal coordinate. SUS represents the diameter of the stainless-steel wire, and PMMA represents the thickness of the acrylic coating. The graph shows that the temperature distribution on the surface of the wire has a lower temperature gradient than that of the airflow. This can be attributed to heat conduction in the wire. The wire surface was heated at the high-temperature side and cooled at the low-temperature side by heat transfer from the airflow. The heat received by the wire on the high-temperature side is transferred to the low-temperature side by heat conduction and then released from the low-temperature side into the airflow. Therefore, the low-temperature side becomes hotter than the airflow temperature, and the high-temperature side becomes cooler than the airflow temperature. This results in a gradual temperature gradient regardless of the wire type. A comparison of the temperature gradient of the airflow for each wire is presented below. Wire ① is the farthest from the temperature distribution of the airflow, and wires ② and ③ are closer to the temperature distribution of the airflow as the thickness of the acrylic increases. This is because the amount of heat transfer inside the wire is proportional to the thermal conductivity, and the decrease in the thermal conductivity of the ultrathin wire due to the increase in the thickness of the acrylic reduces the heat transfer from the high-temperature side to the low-temperature side. Therefore, lowering the thermal conductivity of the wire leads to improved temperature measurement accuracy. Thus, it is important to select a material with low thermal conductivity when manufacturing ultrafine thermosensitive fluorescent wires.

**Figure 9:**
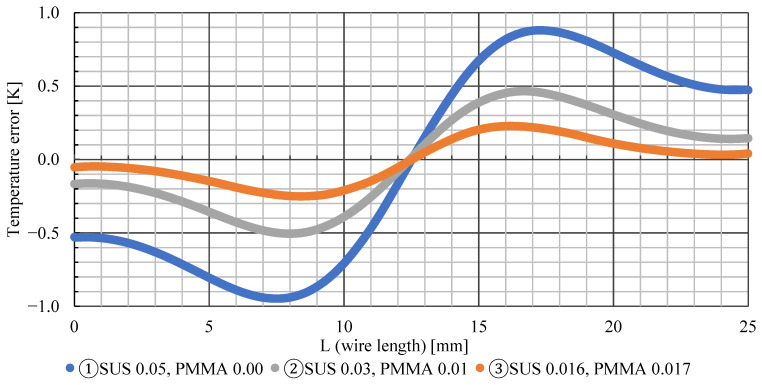
Temperature error.

**Figure 10:**
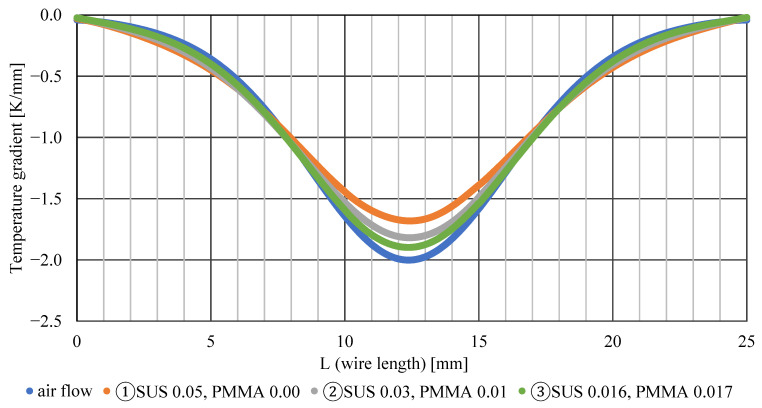
Temperature gradient.

Figure 9 shows that the temperature errors of wires ①, ②, and ③ are ±0.95 K, ±0.52 K, and ±0.26 K, respectively. The smaller the thermal conductivity of the wire, the lower the overall temperature error. Comparing Figure 9 and Figure 10, the temperature error is maximum at the position where the values of the temperature gradient of the wire surface and airflow coincide. Furthermore, the positive and negative temperature errors switch at *L* = 12.5 mm, where the temperature gradient of the wire changes from decreasing to increasing. It is reasonable and obvious that there is a point where the temperature error is exactly zero, owing to the balance between the effects of cooling and heating. Such a zero-error point exists even if the temperature distribution is asymmetric. In this study, we decided to adopt method ② because it is easy to paint and has a small measurement error.

### 4.3. Composition of the Uncertainty in Temperature

The total uncertainty in temperature is defined by the following equation:

(9) $$T_{c}^{2}\left(x\right)=\overset{N}{\underset{i=1}{\sum }}T_{}^{2}\left(x_{i}\right)$$

where *N* is the number of uncertainty factors. That is, by multiplying, *T*(*x_i_*) is each uncertainty factor. The uncertainty originates from a thermocouple is ±0.05 K (bias error). A uniform temperature field was generated by heating and mixing the atmosphere in the test section using a heater and an electric fan. The result indicates that the standard deviation of temperature was ±0.30 K (precision error). Using this equation, the composite uncertainty was evaluated as 0.60 K.

## 5. Application to Flow Field

**Figure 11:**
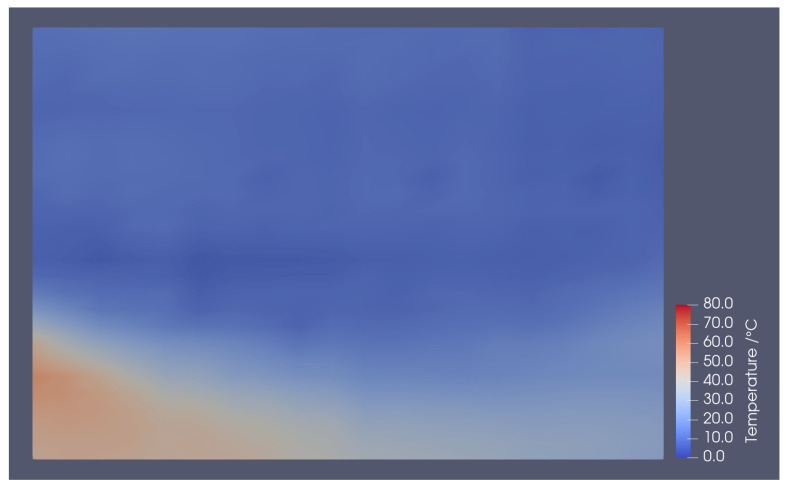
Temperature distribution of mixing layer of heating/cooling airflow.

Figure 11 shows the temperature distribution of the mixing layer of the heating/cooling airflow. This shows that the heating/cooling layers were well visualized and the temperature field was well analyzed. These fluorescent wires did not oscillate, and vortices were observed during the experiment. The flow field was not disturbed, indicating temperature. In future studies, the line profile will be extended to three dimensions using multiple wires. The number of wires required for measuring the three-dimensional temperature distribution was lower than that required when using thermocouples.

Experimental results for a higher temperature range using BAM-B or BAM-G phosphor (100–750 °C) will be reported in a future study.

## 6. Conclusions

We proposed a temperature measurement method that uses ultrafine fluorescent wires to reduce the wire diameter to a considerably lesser extent than that of a thermocouple. On comparing the temperature error between the ultrafine wire and thermocouple, the temperature distribution measurement method using the ultrafine wire was found to be highly accurate and reliable for practical use. This measurement method was applied to measure the temperature distribution of the mixing layer of a heating/cooling airflow.

## Data Availability

Not applicable.
